# Clinical and radiographic outcomes of the SMR TT system – a novel convertible all-polyethylene glenoid with hybrid fixation in anatomic total shoulder arthroplasty: minimum 5-year follow-up

**DOI:** 10.1016/j.jsea.2026.100022

**Published:** 2026-04-21

**Authors:** Agneish Dutta, Idah Chatindiara, Nicholas Buckley, Ryan Gao, Peter C. Poon

**Affiliations:** Department of Orthopaedic Surgery, North Shore Hospital, Auckland, New Zealand

**Keywords:** Hybrid glenoid, Convertible glenoid, SMR, Anatomic shoulder arthroplasty, Total shoulder arthroplasty, Revision shoulder arthroplasty

## Abstract

**Background:**

Modern anatomic total shoulder glenoid implants must achieve sound fixation, low rate of revision, and ease of conversion to a reverse shoulder replacement should the clinical need arise. This study presents the medium term follow-up of a consecutive series of patients being treated for primary glenohumeral joint osteoarthritis with a hybrid convertible glenoid implant.

**Methods:**

This was a retrospective review of a prospective series of patients treated with an anatomic total shoulder replacement with the SMR TT Hybrid Glenoid Implant (LimaCoporate, San Daniele del Friuli). The inclusion criterion was a minimum of 5 years follow-up. Patients were excluded from this review if they had less than 5 years of follow-up. Clinical outcomes were assessed using the American Shoulder and Elbow Surgeons score and the Oxford Shoulder Score, while range of motion was measured using a goniometer. Standardized Grashey and axillary radiographs were analyzed for evidence of implant loosening or component failure.

**Results:**

Forty-two consecutive aTSA performed in 41 patients who received an SMR TT Hybrid Glenoid at a single center between August 2017 and June 2020. There were 24 female patients, and mean age was 68 years (standard deviation: ±8). The mean follow-up duration was 69 months (standard deviation: ±13). The mean pre-operative and post-operative American Shoulder and Elbow Surgeons scores were 27 ± 12 and 87 ± 15, respectively (*P*< .001). The mean pre-operative and post-operative Oxford Shoulder Score scores were 20 ± 7 and 44 ± 5, respectively (*P*< .001). There were significant improvements in range of motion, with mean forward flexion increasing from 71 ± 35 to 144 ± 25 and abduction 68 ± 34 to 134 ± 30 (*P*< .001). Radiographically, 11% showed radiolucent lines affecting the glenoid and 24% affecting the humerus, all asymptomatic. There were no cases of component failure. One patient was revised to a reverse total shoulder arthroplasty for subscapularis failure secondary to trauma.

**Conclusion:**

Our study demonstrates that patients treated with an anatomic total shoulder arthroplasty with SMR TT Hybrid Glenoid had excellent clinical and radiological outcomes at a minimum of 5-year follow-up.

Anatomic total shoulder replacement remains the ‘gold standard’ surgical treatment for patients with symptomatic end-stage glenohumeral joint osteoarthritis with an intact and functioning rotator cuff.^6^^,^^10^^,^^15^ In a small proportion of patients, however, they can fail, with a recent meta-analysis finding that 26% of failures were due to glenoid component loosening (the most common cause of failure in both the early- and later-failure groups).^11^ Rotator cuff insufficiency the next most common cause (17%),^11^ although some studies have found rotator cuff failure to be the most prevalent cause of failure.^17^^,^^23^ Revision for either of these indications is to reverse total shoulder arthroplasty (rTSA) and can be a challenge due to glenoid bone loss or removal of a well-fixed glenoid component, with high complication rates reported.^19^^,^^21^

The ideal glenoid implant should have secure, robust fixation, and the ability for conversion to a reverse component should the need arise. This is the rationale of the SMR TT Hybrid Glenoid (LimaCorporate, San Daniele del Friuli), which uses a polyethylene baseplate and a central Trabecular Titanium (TT) peg, with 2 peripheral pegs that are cemented into the glenoid for rotational stability (Fig. 1). The cemented pegs contribute to primary implant stability, with the central TT peg designed to biologically integrate the implant subsequently. It allows conversion to reverse total shoulder replacement, with dedicated instruments to remove the polyethylene baseplate while retaining the TT peg *in situ*. A reverse baseplate and glenosphere may then be connected onto the central peg. In this way, iatrogenic glenoid bone loss is minimized (Fig. 2).^3^ There are 3 different sizing options for the glenoid baseplate (small, standard, and large), each of which have 2 options for radii of curvature (standard and low mismatch) and 2 thickness options, aiding the ability to achieve soft tissue balancing intraoperatively.

**Figure 1:**
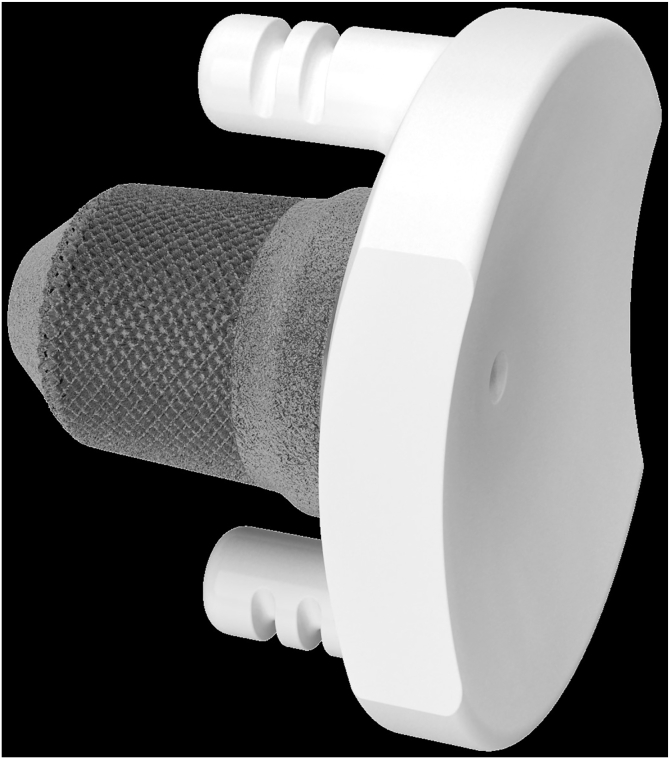
SMR TT Hybrid Glenoid. The SMR TT Hybrid Glenoid with a polyethylene baseplate connected to a central peg made of TT. The baseplate has 2 peripheral pegs intended to be cemented into the native glenoid. Three polyethylene baseplate sizes are available (small, standard, and large), and each size has 2 radii of curvature options (standard and low mismatch) and 2 thicknesses (standard and +2). *TT*, Trabecular Titanium.

**Figure 2:**
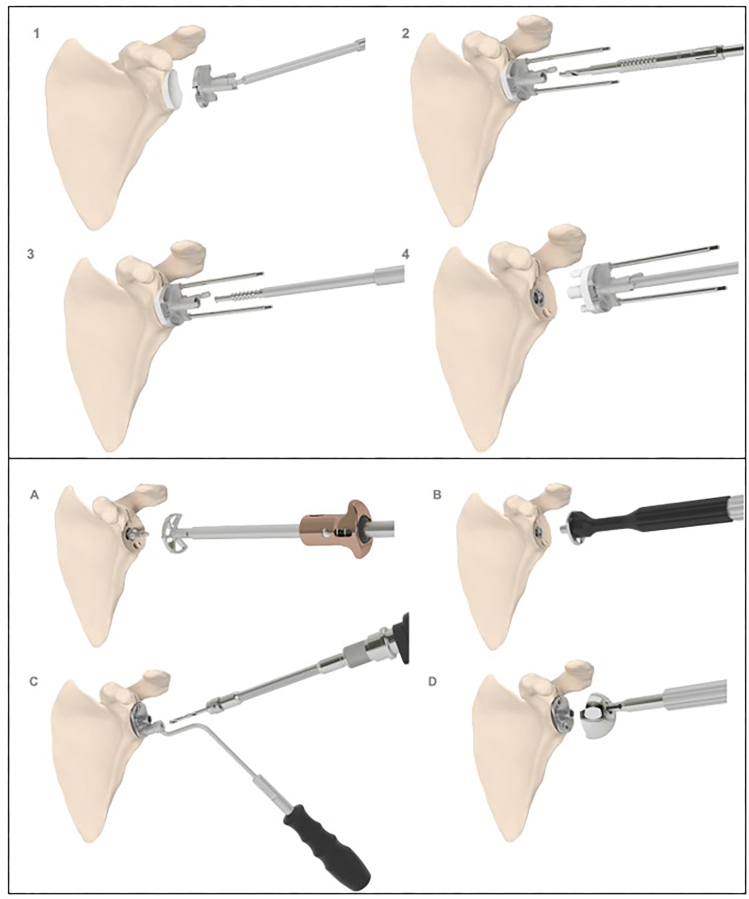
Revision from aTSA to RSA with retention of central TT peg. (1-4) demonstrates explant of the polyethylene baseplate using proprietary instruments without the need for explant of the well-fixed central TT peg. (**A-D**) demonstrates the process of preparation and implantation of the SMR RSA baseplate to the retained TT peg and implantation of the glenosphere. *aTSA*, anatomic total shoulder arthroplasty; *RSA*, reverse shoulder arthroplasty; *TT*, Trabecular Titanium.

While early results using the SMR TT Hybrid glenoid have been promising,^8^ medium term results beyond 5 years remain limited. This study presents the same consecutive series of patients treated for primary glenohumeral joint osteoarthritis using the SMR TT Hybrid glenoid with a minimum 5-year follow-up to assess whether fixation remains reliable in the medium term and the good early clinical outcomes are maintained.

## Materials and methods

### Study design and patient selection

This is a single center prospective study with consecutive patients from New Zealand (district general hospital, secondary care setting, mixture of public, and private cases) treated with anatomic total shoulder arthroplasty (aTSA) using the SMR TT Hybrid Glenoid. Institutional review board approval was granted prior to commencement of the study (registration number RM14180). Patients were identified from a secure clinical database. Inclusion criteria included a minimum 5-year post-operative patient-reported outcome measures using the Oxford Shoulder Score (OSS), overall satisfaction, pain score, and the American Shoulder and Elbow Surgeons (ASES) score. Demographics, including gender, age at time of procedure, and body mass index, were recorded. Range of motion (ROM) was also recorded pre-operatively and post-operatively. Radiological outcomes were assessed from standardized plain-film radiographs (Grashey and axillary lateral views).

A total of 55 primary aTSA procedures using the SMR TT Hybrid Glenoid were performed during the recruitment period (August 2017 to June 2020) in 54 patients, with one patient receiving bilateral procedures. Patients were excluded if they did not complete a minimum 5-year follow-up. Rigorous follow-up procedures were implemented with the goal of including all consecutive cases in the final analysis. Thirteen participants were excluded: 4 were deceased, 4 were lost to follow-up due to being uncontactable or having relocated, 3 declined clinic review reporting their shoulder was asymptomatic, and 1 was unable to attend follow-up due to dementia. This left a final sample of 42 shoulders from 41 participants for analysis.

All included patients completed clinical and functional outcome assessments, including ROM, at the 5-year follow-up. Radiographic assessments were available for 38 of the 42 shoulders in which optimal radiographs were obtainable, and these were used for the radiographic analysis.

### Surgical technique and perioperative care

Four fellowship-trained surgeons performed the operations. All patients received a general anesthetic, interscalene brachial plexus block, and pre-operative and post-operative prophylactic intravenous antibiotics. They were all given 500 mg oral tranexamic acid 3 times daily for 48 hours (a protocol developed following use in knee arthroplasty cases). A deltopectoral approach was used, with patients in the beach-chair position. All patients had an intact rotator cuff. The biceps was tenodesed. The subscapularis was detached by tenotomy, peel, or osteotomy according to surgeon preference. Humeral osteophytes were removed, and the canal was prepared for either an uncemented stemmed or stemless prosthesis (LimaCorporate, San Daniele del Friuli) as per surgeon's preference.

All patients received an SMR TT Hybrid Glenoid. The appropriately sized implant was applied following exposure of the glenoid, soft tissue releases, and reaming, with trial implants used to ensure impingement was avoided and a full ROM achieved on the table. The TT peg was impacted, and the peripheral pegs cemented with Simplex P with Tobramycin (Stryker, Kalamazoo, MI). Following implantation of the components, the subscapularis was repaired with nonabsorbable suture (either tendon-to-tendon, or transosseous) with the use of an endobutton (Smith & Nephew, Andover, MA). Post-operatively, patients were immobilized in a sling with an abduction pillow for 6 weeks, during which only elbow and wrist movements, plus shoulder pendulum movements were permitted. Patients were instructed to avoid external rotation past neutral. After 6 weeks, acute and passive free ROM was worked on with the input of physiotherapists, with strengthening beginning at 3 months post-operatively.

### Outcome measures

#### Clinical outcome measures

Baseline data were collected within 1 month prior to surgery. Clinical and radiographic evaluation took place at 6 weeks, 3 months, and 12 months post-operatively, then annually thereafter.

OSS and ASES scores were recorded, as well as satisfaction (on a 5-point Likert scale) and pain scores (visual analog scale). ROM was measured in a standardized manner as per previous publications^2^^,^^8^ using a goniometer, including shoulder flexion, abduction, and external rotation with elbows at the side. Internal rotation was graded based on the anatomical location reached by the top of the thumb.

#### Radiological outcome measures

Grashey and axillary lateral views were taken at each follow up appointment. Two surgeons (AD and NB) not involved in the operative procedures, independently assessed the radiographs. Initial scores were compared, and cases with discrepant assessments were identified. Agreement between assessors prior to consensus exceeded 90% across all radiological parameters. Discrepancies were subsequently reviewed jointly, with consensus reached through discussion and, where required, consultation with a senior reviewer.

**Figure 3:**
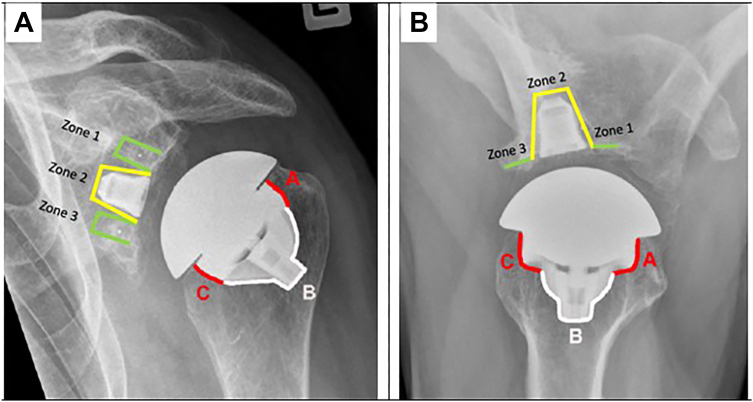
Assessment of radiolucent lines around SMR TT Hybrid Glenoid and SMR stemless anatomic humeral components. (**A**) demonstrates the radiolucent zones in the Grashey view for the SMR TT Hybrid Glenoid and SMR stemless humeral implants, respectively. (**B**) demonstrates the radiolucent zones in the axillary view for the SMR TT Hybrid Glenoid and the SMR stemless humeral implants, respectively.

Assessment for radiolucent lines around the glenoid component was performed by dividing the Grashey-view radiographs into 3 zones (as per Gao et al^8^) (Fig. 3). Zone 1 was the superior cemented polyethylene plug, zone 2 the central TT peg, and zone 3 the inferior cemented plug. In the axillary view, the 3 zones corresponded to the area anterior, around, and posterior to the central peg, respectively. Loosening was graded as per the Lazarus scoring system^12^ (Table I).

**Table I:** Scoring system for glenoid component loosening.

Grade	Findings
0	No radiolucency
1	Incomplete radiolucency around 1 or 2 pegs/screws
2	Complete radiolucency (≤2 mm wide) around 1 peg only, with or without incomplete lucency around 1 other peg
3	Complete radiolucency (≤2 mm wide) around 2 or more pegs
4	Complete radiolucency (>2 mm wide) around 2 or more pegs
5	Gross loosening

Adapted from Lazarus et al.^12^

For the stemless humeral components, assessment was made as described by Albers et al and used by Gao et al^2^^,^^8^ Three zones were used in the Grashey and axillary views; zone A on the Grashey view is adjacent to the cranial part of the proximal ring, B at the fins and central peg, and C at the caudal part of the proximal ring. This is similar on the axillary view. Gruen zones, modified for the humerus, were used to describe location of loosening for the stemmed components.^14^ This involves 7 zones and grading by width (<2 mm or ≥2 mm). Loosening was defined as displacement of the humeral component (by ≥ 2 mm) when comparing the most recent radiograph to the initial post-operative radiograph, or by radiolucency ≥2 mm in more than 3 zones. Subsidence was measured by change (≥2 mm) from the superior aspect of the humeral component and greater tuberosity.

Reduced bone density, particularly at the calcar, was noted to determine presence of stress shielding. Where present, post-operative proximal migration of the humeral head was recorded, and those cases were examined to check if patients were symptomatic or required any nonoperative or operative management as a result.

#### Statistical analysis

Data were analyzed using Statistical Package for the Social Sciences, version 29.0 (IBM, Armonk, NY). Descriptive statistics were calculated for all variables. Continuous variables are reported as mean ± standard deviation and categorical variables as frequencies and percentages. Missing data were minimal: 11 patients lacked preoperative clinical scores, for which mean imputation was applied. No other data imputation procedures were performed. Clinical and functional scores at pre-operative and ≥5 years post-operative were compared using a paired *t*-test. Significance tests were two-tailed, with a significance level of *P*< .05 used. To account for multiple comparisons across clinical and functional outcomes (OSS, satisfaction, pain, ASES, and 4 ROM variables), a Bonferroni correction was applied. With 8 comparisons performed, the adjusted significance threshold was set at *P*< .00625. All reported *P* values remained <.001 and were therefore statistically significant after correction.

## Results

### Demographics

A total of 42 aTSAs were performed in 41 patients (Table II). Of these, 24 (57%) were females and 18 (43%) males. The mean age at the time of surgery was 68 ± 8 years, with a mean body mass index of 31 ± 6 kg/m^2^. The mean American Society of Anesthesiologists score was 2 ± 1. All patients had a primary diagnosis of glenohumeral osteoarthritis. A stemmed humeral component was used in 26 cases (62%) and a stemless design in 16 cases (38%). The mean follow-up duration was 69 ± 13 months.

**Table II:** Baseline data (N = 42).

Category	Number %
Gender	
Female	24 (57%)
Male	18 (43%)
Age at procedure (yr)	
Mean ± SD<	68 ± 8
BMI (kg/m^2^)	
Mean ± SD	31 ± 6
ASA score	
Mean ± SD	2 ± 1
Primary diagnosis	
Osteoarthritis	42 (100%)
Humeral Stem	
Stem	26 (62%)
Stemless	16 (38%)
Follow-up duration for survival analyses (mo)	
Mean ± SD	69 ± 13

*ASA*, American Society of Anesthesiologists; *SD*, standard deviation; *BMI*, body mass index.

Number (%) reported unless mean ± SD is specified.

All percentages reported to the nearest integer.

### Clinical and functional outcomes

At a mean follow-up of 69 months, patients demonstrated significant improvements across all patient-reported outcome measures (Table III). The mean OSS increased from 20 ± 7 pre-operatively to 44 ± 5 post-operatively (mean change = 24 [95% confidence interval: 21.0-26.6], *P*< .001). The mean ASES score improved from 27 ± 12 to 87 ± 15 (mean change = 60 [95% confidence interval: 54.0-65.4], *P*< .001).

**Table III:** Clinical and functional outcomes (§N = 41).

Clinical or functional outcome measure	Pre-operative scores (ROM in degrees)	Post-operative scores (ROM in degrees)	Mean change [95% CI] (post-operative *minus* pre-operative scores)	∗*P* value
Oxford shoulder score (OSS)	20 ± 7	44 ± 5	24 [21,0-26.6]	<.001∗
Overall satisfaction†^.^	1 ± 1	5 ± 1	3 [2.7-3.4]	.001∗
Pain score	8 ± 1	1 ± 1	−7 [−7.1 to −5.9]	.001∗
American shoulder and Elbow score (ASES)	27 ± 12	87 ± 15	60 [54.0-65.4]	.001∗
ROM: forward flexion	71 ± 35	144 ± 25	71 [57.5-86.2]	.001∗
ROM: abduction erect	68 ± 34	134 ± 30	65 [51.0-80.0]	.001∗
ROM: external rotation	30 ± 17	44 ± 17	14 [6.4-22.3]	.001∗
ROM: internal rotation‡^.^	1 ± 1	4 ± 1	2 [1.9-2.6]	.001∗

*CI*, confidence interval; *ROM*, range of motion; *PROM*, patient-reported outcome measure; *SD*, standard deviation.

All values reported as mean ± SD. All mean values reported to the nearest integer; 95% CI reported to 1 d.p.

∗ *P* value significant at *P*<.*050*, Paired t-test.

† Overall satisfaction (1-5) based on patient report—1, very dissatisfied, 2 = dissatisfied, 3 = neutral, 4 = satisfied, 5 = very satisfied.

‡ ROM: internal rotation: (1-5) based on ability to reach 1 = hip, 2 = sacroiliac joint, 3 = midlumbar, 4 = thoracolumbar joint, 5 = midthoracic.

§ Note: analyses were based on 41 cases, as the 5-yr PROM and ROM data from the revised case were excluded from the final outcome scores analyses.

Pain and satisfaction also improved substantially, with mean pain scores decreasing from 8 ± 1 to 1 ± 1 (*P*< .001) and overall satisfaction increasing from 1 ± 1 to 5 ± 1 (*P*< .001). ROM demonstrated significant improvements: mean forward flexion improved from 71° ± 35° to 144° ± 25°, abduction from 68° ± 34° to 134° ± 30°, external rotation from 30° ± 17° to 44° ± 17°, and internal rotation (graded 1-5) from 1 ± 1 to 4 ± 1 (all *P*< .001 remaining statistically significant after Bonferroni correction) (Table III).

### Radiological outcomes

Thirty-eight of the 42 shoulders (91%) had optimal post-operative radiographs available for a minimum 5-year follow-up. There were no cases of component failure in the study period. Four of 38 cases (11%) showed grade 4 glenoid radiolucency (2 in zones 1-3, 1 in 1 and 3, 1 in zone 2). Nine of 38 (24%) showed humeral loosening, most in the greater tuberosity or calcar region. Of these 9 cases, 6 were stemless implants. Subsidence was observed in 1 case (3%). Stress shielding was observed in ten of 38 shoulders (26%), predominantly in the calcar region. Superior migration was noted in ten of 38 shoulders (26%); however, 90% of these patients had no clinical symptoms or required any operative management. The 1 of 10 (10%) who did report pain with superior migration was managed nonoperatively.

### Complications and revisions

One complication was identified. This was a subscapularis failure, which occurred just under 5 years after the primary arthroplasty procedure, secondary to trauma. The last radiographs showed no evidence of component loosening, subsidence, or stress shielding. The patient was revised to a reverse total shoulder replacement. There were no cases fracture or infection.

**Figure 4:**
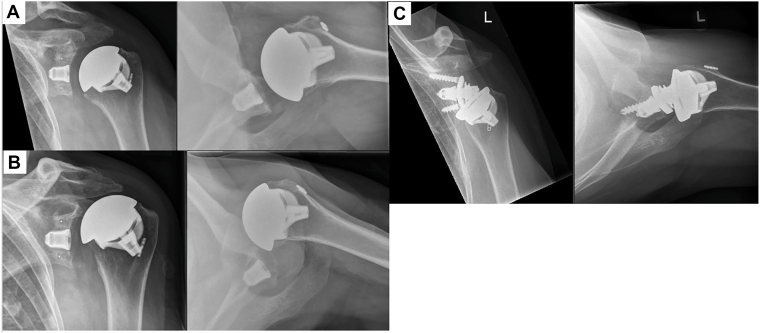
Case revised from anatomic to reverse total shoulder replacement. (**A**) demonstrates the post-operative imaging following the index procedure. (**B**) demonstrates the anterior humeral translation following subscapularis failure. (**C**) demonstrates the revision to reverse total shoulder replacement.

In the revision case, the patient was found to have a thinned subscapularis tendon, the remnants of which were detached from the humerus, with an associated supraspinatus tear. The humeral head and morse taper were removed. The polyethylene insert was easily removed from the glenoid, and the central TT peg remained, with bony ingrown evident. A baseplate was then implanted and fixed with 2 screws, followed by implantation of the glenosphere and cup. Good soft tissue tension and stability were confirmed. Closure was achieved after thorough wash with betadine, hydrogen peroxide, and antibiotic mixed with saline. The remnant of the supscapularis tendon was repaired, and vancomycin powder was applied. At 12 months follow-up, the patient reported a visual analog scale pain score of two of 10, Stanmore Percentage of Normal Shoulder Assessment of 95%, a full range of forward flexion, and 45° external rotation, with no further complications (Fig. 4).

## Discussion

This prospective study demonstrates excellent medium-term clinical and radiological outcomes using the SMR TT hybrid glenoid. The implant retention rate at a minimum of 5 years was 100%. None of the glenoid implants failed, and there were no cases of symptomatic glenoid or humeral component loosening. Of the cases showing radiolucent lines, majority were around stemless implants. They may be explained by initial micromotion or bone remodeling. There are limitations in the interpretation of reduced bone density in radiographs due to differences in contrast and overlying soft tissue potentially causing changes in appearance. It should be noted that none of these cases were symptomatic; however, should be followed up in the longer term. There was one revision due to subscapularis failure. The anatomic prosthesis was easily converted to a reverse total shoulder replacement, and the patient subsequently did well functionally, with no further complications. This adds to the existing literature supporting the ease of conversion from aTSA to rTSA using the SMR TT Hybrid Glenoid.^3^

aTSA has increasingly been used for shoulder osteoarthritis and has demonstrated favorable outcomes even in older age groups.^5^^,^^22^ Historically, glenoid loosening has been identified as a major cause of failure, particularly with cemented all-polyethylene components in the longer term.^18^ Issues with all-polyethylene glenoid components include backside wear, debris, and glenoid bone loss during revision surgery.^20^ As a result, metal-backed glenoids were developed, aiming to achieve osteointegration over time and reduce component failure rates. However they demonstrated higher complication rates than the ‘standard’ cemented polyethylene glenoid implants.^9^^,^^18^ They were found to fail for reasons such as component fracture, dissociation, and wear (both of metal or polyethylene); accelerated polyethylene wear and associated proximal humerus osteolysis, implant loosening, glenoid bone loss, and soft tissue deficiency have also been described in the literature.^4^ Boileau et al^4^ found that metal-backed trays can rarely be preserved for reinsertion of a polyethylene insert due to bone loss, negating one of the perceived advantages of the metal-backed glenoid.

The development of a hybrid system aims to avoid the complications associated with both all polyethylene and metal-backed glenoids. They use a central press-fit metal insert with peripheral polyethylene pegs. Design of glenoid components is improving, and material properties with regard to wear resistance are improving. A recent systematic review found that the most common cause for revision of a failed aTSA to rTSA was rotator cuff pathology, with subsequent instability and loosening.^1^ Page et al^16^ found in their study from the Australian National Joint Registry that the most common cause of revision for uncemented glenoid aTSA to rTSA was rotator cuff insufficiency. The next step in implant design is to have a well-fixed glenoid that is also easily convertible. Marigi et al found in their literature review that hybrid glenoid components with a central porous titanium post demonstrated lower rates of radiolucent lines and failures when compared to all-polyethylene glenoid components at up to 5 years.^7^^,^^13^

The SMR TT Hybrid Glenoid is both hybrid and fully convertible to rTSA. In the revision setting, the polyethylene insert is easily detachable, leaving the central TT peg, which is well-fixed, integrated into the native glenoid bone. A reverse baseplate and glenosphere may then be attached to this peg, resulting in no unnecessary bone loss.

This study shows excellent results at a minimum of 5 years post-operatively. Only one revision was identified in our series as a result of rotator cuff failure secondary to trauma, with good glenoid fixation still present at time of revision to rTSA.

### Strengths and limitations

This study has several strengths but also important limitations. The sample size is modest, and no comparative control group was included, precluding direct comparison with alternative glenoid designs. In addition, not all patients implanted during the recruitment period were available for analysis at the minimum 5-year follow-up. However, the observed attrition is consistent with expectations in an older, consecutive shoulder arthroplasty population over this timeframe. Only a small number of participants were truly lost to follow-up; the remaining exclusions were due to death, relocation, or inability to attend review for clear medical reasons, often with confirmation that the operated shoulder was asymptomatic. All consecutive cases within the recruitment period were considered for inclusion, and the analyzed cohort reflects the majority of the original series, minimizing selection bias and demonstrating the rigor of the follow-up process.

A further limitation is that this study focused on a single implant, reducing its generalizability to aTSA cases using different implant designs. The senior author in the study receives royalties from use of the implant, which is a source of potential bias.

The cohort was sufficient to demonstrate statistically significant and clinically meaningful improvements. The presence of both pre-operative and post-operative score enable a more robust within-patient assessment of the implant performance, and the minimum 5-year follow-up enhances the reliability of the findings. This study of clinical and radiographic outcomes adds to existing literature, which is scarce for comparable implants at 5 years of follow-up or more. Further multicenter studies with extended follow-up duration would be beneficial to report on the performance and survival of the implant.

## Conclusion

Patients treated with aTSA using the SMR TT Hybrid Glenoid demonstrated excellent clinical and radiographical outcomes at a minimum of 5 years follow-up. The unique feature of its convertibility allows ease of subsequent conversion of anatomic to reverse configuration.

## Disclaimers:

Funding: The study was funded by LimaCorporate.

Conflicts of interest: Ryan Gao is a consultant for LimaCorporate.

Peter C Poon receives patent fees for the SMR TT Hybrid Glenoid from LimaCorporate. He is a consultant for LimaCorporate.

Patient consent: Obtained.

## Data availability statement

The data that support the findings of this study are available on request from our institution research center - research@waitematadhb.govt.nz. The data are not publicly available due to privacy or ethical restrictions.
